# Crystallization of Ge_2_Sb_2_Te_5_ thin films by nano- and femtosecond single laser pulse irradiation

**DOI:** 10.1038/srep28246

**Published:** 2016-06-13

**Authors:** Xinxing Sun, Martin Ehrhardt, Andriy Lotnyk, Pierre Lorenz, Erik Thelander, Jürgen W. Gerlach, Tomi Smausz, Ulrich Decker, Bernd Rauschenbach

**Affiliations:** 1 Leibniz Institute of Surface Modification, Permoserstr. 15, D-04318 Leipzig, Germany; 2 MTA-SZTE Research Group on Photoacoustic Spectroscopy, University of Szeged, Dóm tér 9, H-6720 Szeged, Hungary; 3 Institute for Experimental Physics II, Leipzig University, Linnéstr. 5, D-04103 Leipzig, Germany

## Abstract

The amorphous to crystalline phase transformation of Ge_2_Sb_2_Te_5_ (GST) films by UV nanosecond (ns) and femtosecond (fs) single laser pulse irradiation at the same wavelength is compared. Detailed structural information about the phase transformation is collected by x-ray diffraction and high resolution transmission electron microscopy (TEM). The threshold fluences to induce crystallization are determined for both pulse lengths. A large difference between ns and fs pulse irradiation was found regarding the grain size distribution and morphology of the crystallized films. For fs single pulse irradiated GST thin films, columnar grains with a diameter of 20 to 60 nm were obtained as evidenced by cross-sectional TEM analysis. The local atomic arrangement was investigated by high-resolution Cs-corrected scanning TEM. Neither tetrahedral nor off-octahedral positions of Ge-atoms could be observed in the largely defect-free grains. A high optical reflectivity contrast (~25%) between amorphous and completely crystallized GST films was achieved by fs laser irradiation induced at fluences between 13 and 16 mJ/cm^2^ and by ns laser irradiation induced at fluences between 67 and 130 mJ/cm^2^. Finally, the fluence dependent increase of the reflectivity is discussed in terms of each photon involved into the crystallization process for ns and fs pulses, respectively.

Phase-change materials such as Ge_2_Sb_2_Te_5_ (GST) possess some remarkable properties that have led to extensive studies within the field of optical data storage^1^ and non-volatile memories^2^. A wider range of potential applications has very recently been explored including optoelectronic displays^3^,^4^, optical communication^5^, reconfigurable optical circuits^6^, and computing^7^. The basic idea of the above mentioned data storage applications relies on the reversible transformation between the disordered amorphous and ordered crystalline states, which can be achieved by local heating/cooling (either with laser or electrical pulses) at high rates. The phase transformation is accompanied by changes in optical reflectivity and electrical resistivity contrast between the two solid states and this is used for reading out the stored data^8^. The crystal structure of metastable crystalline GST can for most purposes be approximated by a cubic rocksalt structure^1^,^9^,^10^,^11^. Te atoms occupy one fcc sub-lattice, whereas the other sub-lattice is randomly occupied by Ge and Sb atoms as well as vacancies. A mechanism of fast phase-change was initially proposed with the umbrella-flip model^8^ that reveals a simple switch of Ge atoms between tetrahedral and octahedral symmetry positions during the transformation process, which does not require breaking the strong covalent bonds. However, this is still under discussion regarding ultrafast phase transition processes^12^,^13^.

Starting from an amorphous disordered atomic arrangement, energy is supplied to the system thus increasing the temperature in the material and inducing the phase transformation in GST films. In the case of optical switching, this energy transfer usually is realized by supplying additional heat directly via photon energy transfer to excited electronic states and subsequent relaxation^8^,^14^. Coherent light is used in optical switching to induce the phase transformation. Different experimental studies on the property changes of phase-change materials upon nano-, pico- to femtosecond laser pulse irradiation^15^,^16^,^17^,^18^,^19^,^20^,^21^,^22^,^23^ have been published in the last years. However, comparative examinations are still lacking.

Regarding the physical process of laser-induced phase transition, on the one hand ns laser pulse irradiation can be considered as a thermal process, while upon excitation with ultrashort pulses (fs) non-thermal effects are probable to be involved in the phase transition process^24^,^25^,^26^. On the other hand, the impact of laser fluence on the disorder-order phase transition is critically important with respect to controlling the crystallization process, especially in order to optimize the major issue of energy consumption during encoding information as well as to benefit to the potential application of multi-level storage^27^. An examination of the influence of the laser wavelength on crystallization has shown that the use of ultraviolet ns laser pulses is favorable in terms of minimizing feature sizes and surface roughness of the films^28^. Therefore, it is of great value to compare the process of GST crystallization induced by single laser pulses in the time scale frame of ns and fs at the same wavelength at variation of the laser fluence.

Consequently, in this study the results of crystallization of pulsed laser deposition (PLD)-deposited GST films by ns and fs single pulse laser irradiation at the same wavelength of 248 nm are compared. The details of the crystallization process are demonstrated by investigation of the films structure, morphology and reflectivity as a function of the laser fluence. The crystallization mechanism of GST films in dependence on the laser fluence induced by ns and fs single laser pulses is discussed based on the effect of each laser photon involved in the phase transformation. It is proven that single pulse fs laser irradiation is more effective for the crystallization of GST films than single pulse ns laser irradiation.

## Results and Discussion

### Structure analysis

**Figure 1:**
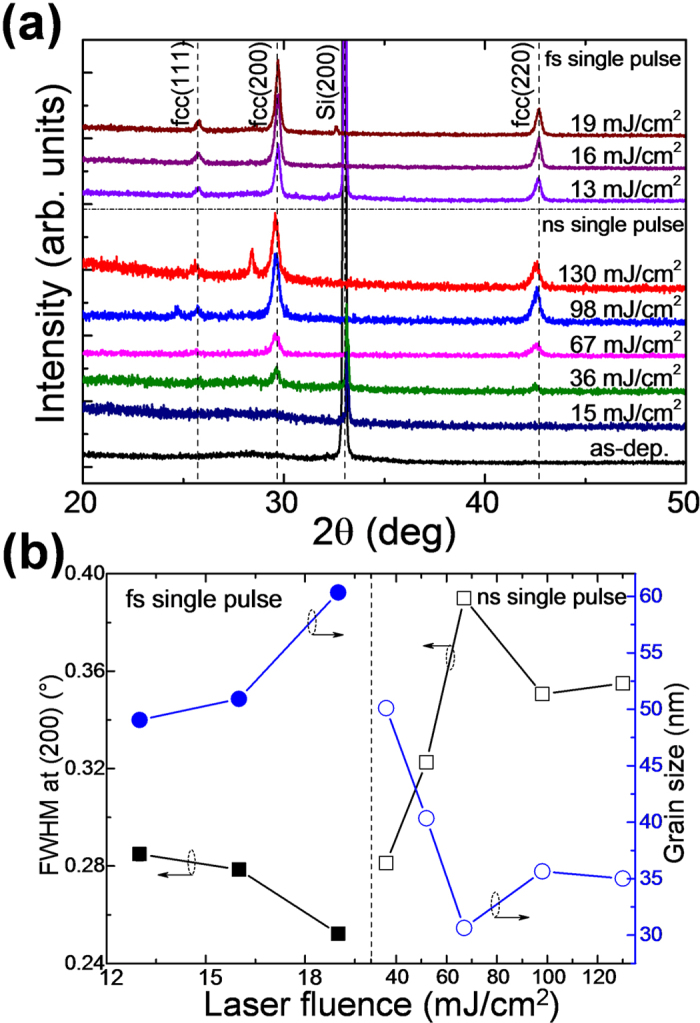
(**a**) XRD spectra of an as-deposited GST film and of films after 500 fs and 20 ns single laser pulse irradiation at different fluences. (**b**) FWHM of the rocksalt phase GST(200) reflection and corresponding calculated average grain sizes as a function of laser fluence (fs and ns denoted by the symbols with interior solid and open, respectively).

X-ray diffraction (XRD) measurements were employed to investigate the influence of the laser fluence on the crystallinity of as-deposited GST films upon single pulse ns and fs laser irradiation. Figure 1a presents the evolution of XRD patterns of films irradiated with increasing laser fluences. The fluence was varied between 0 and 130 mJ/cm^2^ and between 13 and 19 mJ/cm^2^ with pulse durations of 20 ns and 500 fs, respectively. The diffraction pattern of the as-deposited film shows only a small increase of the background located around 28°, which is characteristic of GST in the amorphous state. The diffraction peak at ~33° belongs to Si (200) and therefore is a substrate contribution. For 20 ns laser irradiation with fluences ≥36 mJ/cm^2^ crystalline phase diffraction peaks at 29.6° and 42.7° are visible, which correspond to the (200) and (220) lattice planes of the metastable fcc GST phase, respectively. At laser fluences above 98 mJ/cm^2^, the diffracted intensity is increased and the (111) reflection is visible additionally. It should be noted that although partial crystallization of the films is confirmed from optical reflection measurements (as described in the section dealing with the optical reflectivity) already for fluences between 15 and 36 mJ/cm^2^, the XRD reflections of the crystalline phase cannot be detected due to the low fraction of the crystalline phase in this particular fluence range. With a continuous increase of the laser irradiation fluence from 36 to 98 mJ/cm^2^, the intensity of the peaks became gradually proportional to the volume fraction of the crystalline phase. For the films irradiated with a fs single pulse each, the diffraction spectra are qualitatively the same with the exception that the first diffraction peaks are observed already at a fluence of 13 mJ/cm^2^. A further increase of the laser fluence promotes crystallite growth as evidenced by the increase of the diffracted intensity. The average size of grains in GST films after ns and fs irradiation as a function of laser fluence was estimated from the full width at half maximum (FWHM) of the (200) reflection by using the Debye-Scherrer formula and the results are presented in Fig. 1b. It follows that the average grain size decreases from about 50 nm to 35 nm after single ns pulse laser irradiation in the fluence range between 36 and 130 mJ/cm^2^. As plotted in the left part of Fig. 1b, for GST films after fs pulse laser irradiation the average grain size is observed to gradually increase from about 50 to 60 nm with an increase of the fluence from 13 to 19 mJ/cm^2^. Accordingly, the experiments have shown that a crystallization of GST films is possible in both cases, after ns single pulse and also after fs single pulse laser irradiation for laser fluences >36 mJ/cm^2^ and >13 mJ/cm^2^, respectively. Temperature calculations were carried out by the finite element method using one- and two-temperature models (see Supplementary Section 1). The calculated temperature distribution for layered samples (GST/SiO_2_/Si) shows a distinct maximum within a shallow surface region which exceeds the crystallization temperature of GST. Consequently, a crystallization process in the GST films can be expected.

### Morphology

**Figure 2:**
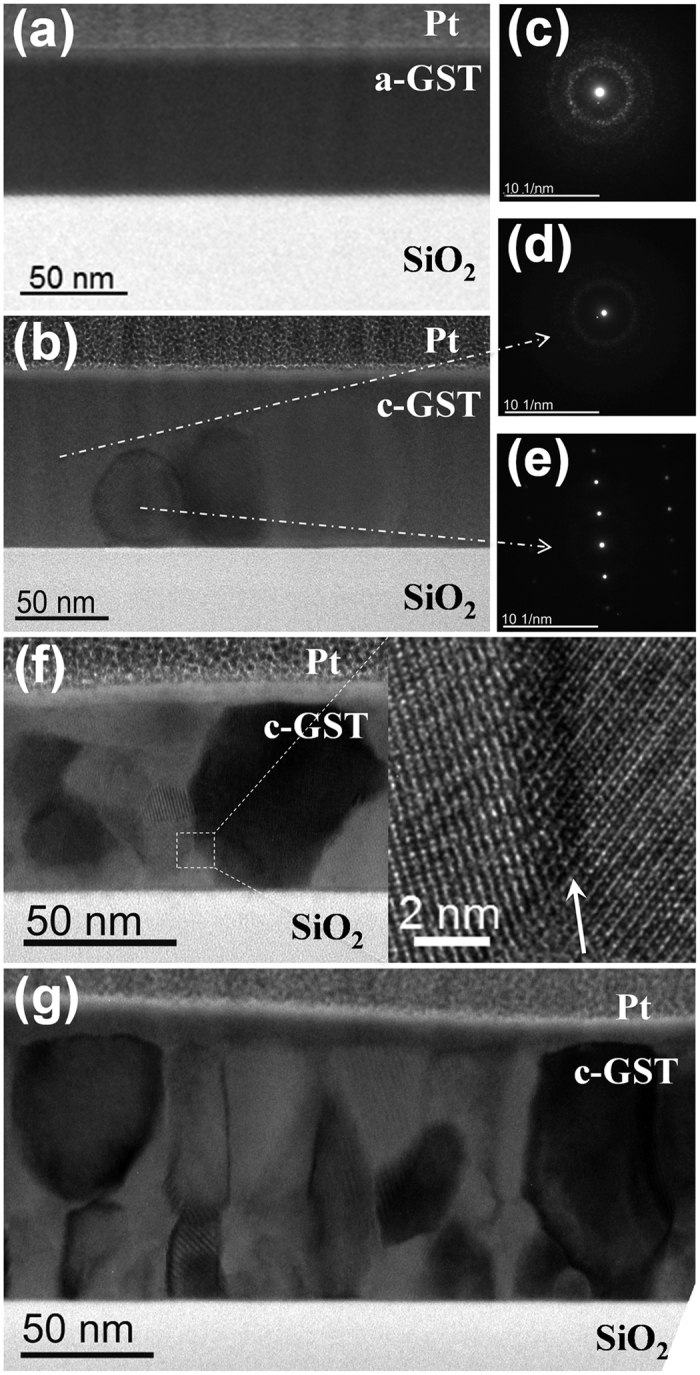
Cross-sectional BF-TEM images and NBD pattern of GST thin films: (**a**) and (**c**) as-deposited; (**b**,**d**,**e**) after ns laser pulse irradiation with 36 mJ/cm^2^; (**f**) after ns laser pulse irradiation with 67 mJ/cm^2^, the insets show magnified regions; (**g**) after fs laser pulse irradiation with 19 mJ/cm^2^.

To understand the crystallite growth process in the GST thin films after ns and fs single pulse laser irradiation, the microstructure evolution was examined by cross-sectional transmission electron microscopy (TEM). Figure 2 comprises cross-sectional bright-field (BF) TEM images and a nanobeam electron diffraction (NBD) patterns of GST thin films before laser irradiation (2a and 2c), after ns single pulse laser irradiation with fluences of 36 mJ/cm^2^ (2b, 2d and 2e) and 67 mJ/cm^2^ (2f) as well as after fs single pulse laser irradiation with a fluence of 19 mJ/cm^2^ (2g). Figure 2a shows a uniform and smooth GST film between the Pt top layer and the SiO_2_ bottom layer, the corresponding NBD pattern in Fig. 2c is typical for an amorphous structure. In contrast, crystalline grains with an oval shape developed within the amorphous GST matrix after laser irradiation by a ns single laser pulse with 36 mJ/cm^2^ (Fig. 2b). With a further increase of the laser fluence to 67 mJ/cm^2^, the crystalline precipitates in the amorphous regions grow to grains with an oval shape filling in the whole film. Between these grains small angle grain boundaries are visible (see Fig. 2(f)). Similarly, even when the laser fluence increases up to 130 mJ/cm^2^ the irradiated GST film occurs to be completely crystalline and the grain diameters in the film vary between 15 and 60 nm. In contrast, the morphology after fs single pulse irradiation of amorphous GST thin films is different to the morphology of GST thin films irradiated by a ns single pulse. Figure 2(g) shows a cross-sectional BF TEM image of the GST thin film after single fs pulse laser irradiation with 19 mJ/cm^2^. A homogeneous polycrystalline structure consisting of columnar grains can be observed with the majority of them extending over the full film thickness of ~90 nm. The diameter of these columnar grains is in the range of 20 and 60 nm.

### Local atomic arrangement

**Figure 3:**
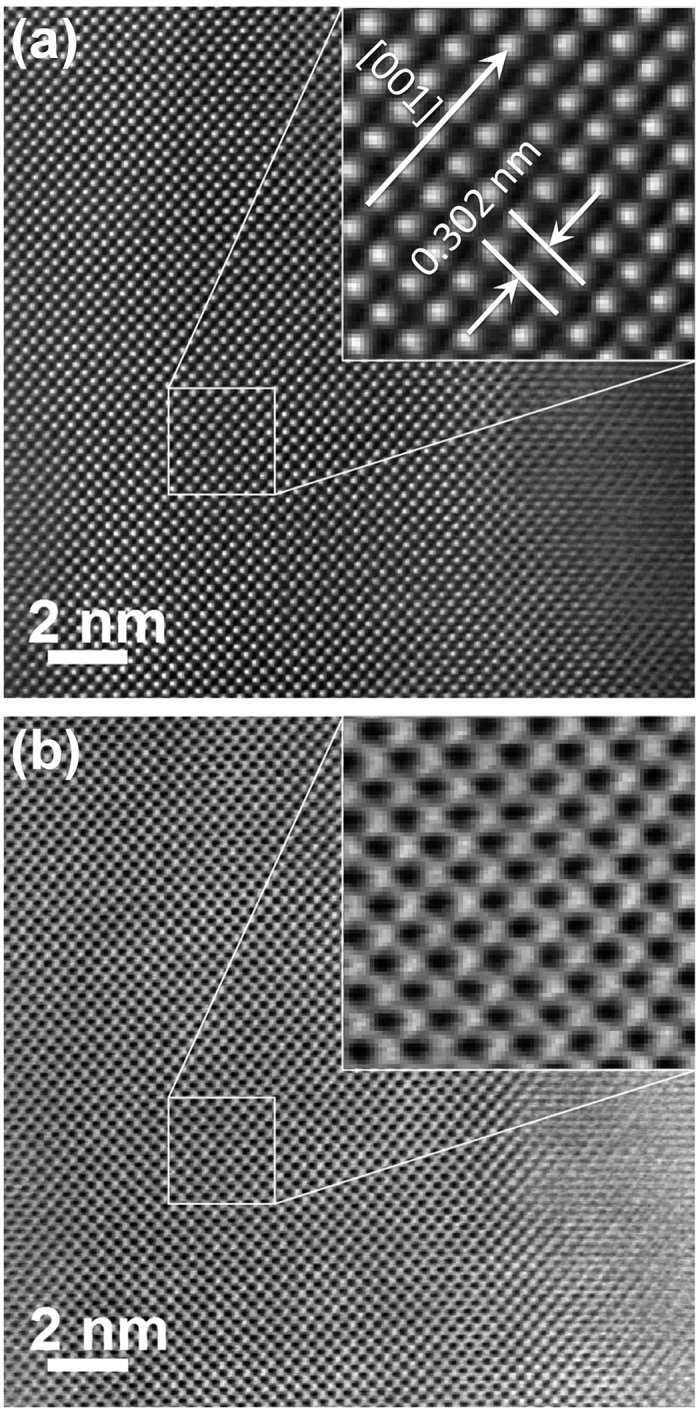
Aberration-corrected (**a**) MAADF- and (**b**) LAADF-HRSTEM images of a representative fs single laser pulse irradiated GST film viewed along the [001] zone axis. The insets show a magnification of the marked region. The corresponding overview image is shown in the Supplementary Section 2 (Fig. S2).

High-resolution scanning transmission electron microscopy (HRSTEM) investigations allow a direct insight into the local atomic arrangement. In Fig. 3a, a middle-angle annular dark-field (MAADF) HRSTEM image along the [001] viewing direction of an as-deposited GST film after single fs laser pulse irradiation is depicted. The real-space lattice fringes in the image indicate that the GST film is characterized by a high crystalline quality. The crystal plane spacing of the film is about 0.302 nm, which corresponds to the {002} crystal plane spacing (0.302 nm) of the cubic GST crystalline phase. It is in reasonable agreement with the results from XRD measurements (the interplanar distance of {002} crystal plane is calculated to be 0.301 nm, according to the Bragg equation). It should be noted, that no additional sublattices^29^ are formed by Ge atoms located at tetrahedral positions^12^ or Ge atoms located at off-octahedral positions^13^ as evident from atomic-resolution low-angle annular dark field (LAADF) HRSTEM studies (see Fig. 3b). This is in contrast to the results of Liu *et al.* claiming a coexistence of octahedral and tetrahedral sites for Ge in the GST lattice^13^. In addition, detailed analyses of local distortions in the GST lattice showed that a distorted octahedral atomic arrangement of GeSb is the main structural motif for the metastable and amorphous GST phases^29^. Thus, due to laser irradiation these distortions of the GST lattice can be even enhanced. This will induce a breaking of long-range order and lead to the transition from the metastable to the stable GST phase as was proposed by Kolobov *et al.*^11^. Another possibility of phase transition is melting of the GST lattice due to lattice heating caused by the transfer of electron energy to the covalent backbone by applying an ultrashort laser pulse^14^. Moreover, a non-thermal amorphization process caused by displacements of Ge atoms from octahedral to tetrahedral sites induced by ultrashort laser pulses was also reported recently^30^.

### Optical reflectivity

**Figure 4:**
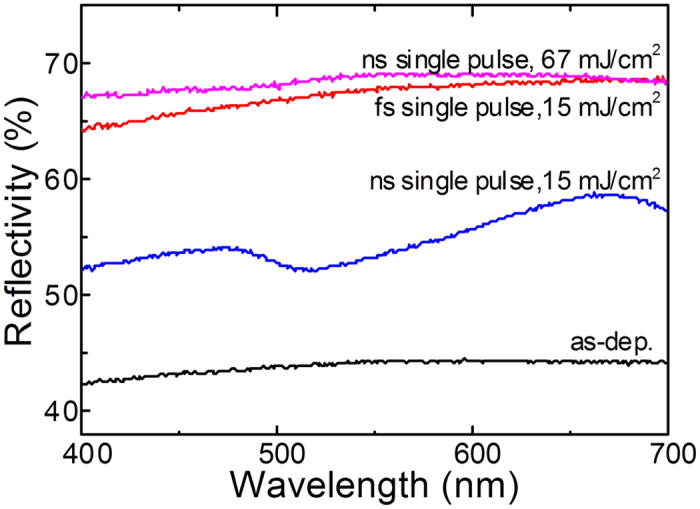
Optical reflectivity of GST films after deposition (as-dep.), after 20 ns single laser pulse irradiation at fluences of 15 and 67 mJ/cm^2^, and after 500 fs single laser pulse irradiation at a fluence of 15 mJ/cm^2^.

Figure 4 shows the reflectivity of GST films deposited at room temperature after 20 ns single pulse laser irradiation with fluences of 15 and 67 mJ/cm^2^, and after 500 fs single pulse laser irradiation with a fluence of 15 mJ/cm^2^. The optical reflectivity of the as-deposited, amorphous GST film is approximately 43% for wavelengths between 400 and 700 nm. In contrast, the reflectivity of the film after ns pulse laser irradiation with a fluence of 15 mJ/cm^2^ ranges between 52 and 58%. With increasing the laser fluence up to 67 mJ/cm^2^, a higher reflectivity in the range from 65 to 67% can be observed. For the fs-irradiated film, the optical reflectivity is in the range from 64 to 67%, which is comparable to that of the ns-irradiated film with a fluence 67 mJ/cm^2^. These results demonstrate that a phase transition from the amorphous to the crystalline state can be assumed by ns single laser pulse irradiation with a fluence ≥15 mJ/cm^2^. With an increase of the laser fluence (67 mJ/cm^2^; see Fig. 4) the optical reflectivity is further increased. This is an indication that the initially small crystalline volume embedded in the amorphous matrix increased. When comparing the reflectivity of the films after ns- and fs-single laser pulse irradiation at a fluence of 15 mJ/cm^2^ (see Fig. 4), it is obvious that single pulse fs-laser irradiation is more effective with regard to the crystallization process. Similar results were also found by XRD characterization (see the section dealing with the structure analysis).

**Figure 5:**
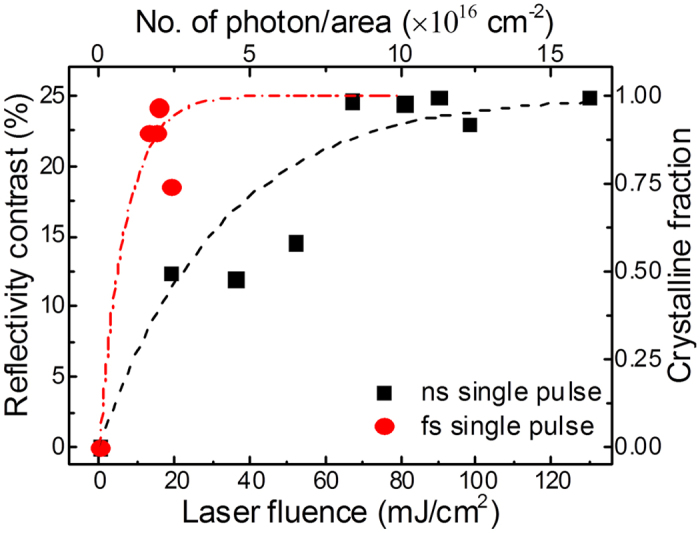
Optical reflectivity contrast of GST films at a wavelength of 650 nm after ns and fs laser pulse irradiation with a single pulse as a function of the laser fluence and the number of laser photons/area.

In Fig. 5 a series of irradiation fluence dependent reflectivity measurements at a chosen wavelength of 650 nm of GST films are shown. As expected, the reflectivity of the films increases with increasing laser fluence, but the reflectivity change after fs-irradiation at a fluence of 15 mJ/cm^2^ is very steep in contrast to that after the ns laser irradiation. A simple rescaling procedure of the laser fluence leads to the number of photons per unit area N_p_


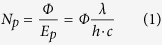


where Φ is the laser fluence, E_p_ the energy of the photons, h the Planck constant, c the speed of light in vacuum and λ the laser wavelength (here 248 nm). The change of the optical reflectivity contrast is proportional to the amount of the crystalline fraction implied by the laser photons^7^,^31^. Consequently, it can be assumed that the crystallization of GST thin films is completed when no change of the optical contrast can be determined anymore as function of the laser fluence. The results of the rescaling procedure are included in Fig. 5.

In the idealized case, it might be assumed that every photon which hits the surface induces crystallization into the surrounding amorphous matrix, resulting in a cylinder-like cluster with a sufficiently large crystalline area a_c_ at the surface. Then, the formation rate of the crystalline fraction with the number of photons per area (laser fluence) can be given by


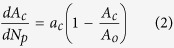


where A_c_ is the area covered by crystalline clusters and A_o_ is the total irradiated area. The integration of this simple differential Equation (2) leads to


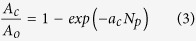


where A_c_/A_o_ represents the crystalline fraction on the total area. Equation (3) was used to fit the experimental data in Fig. 5, where the black (dash) and red (dash dot) lines indicate the crystalline fraction according to Equation (3) after single ns and fs pulse laser irradiation, respectively. Good fits are obtained if the circular areas a_c_ = 0.0025 nm^2^ and a_c_ = 0.0115 nm^2^ are used for ns- and fs-laser irradiation, respectively. These results correspond to the radii of the cylinder-like crystalline clusters of r_c_ = 0.02 nm and r_c_ = 0.043 nm, respectively. The maximum length of the cylinder-like clusters is given by the total absorption of the incident laser light (wavelength is 248 nm and photon energy is about 5 eV). According to the Beer-Lambert absorption law the absorption length can be determined to be 50 nm, if an absorption coefficient of 8 × 10^5 ^cm^−1^ is assumed for crystalline and amorphous GST phases^32^. Consequently, the average volume which is transferred by a single laser photon with a wavelength of 248 nm is 0.125 nm^3^ and 0.575 nm^3^ after ns- and fs-laser irradiation, respectively. Assuming that the atomic density of the amorphous phase is 3.34 × 10^22^ atoms/cm^3^ 33 about 5 and 20 GST atoms participate in the amorphous-crystalline phase transition per photon by ns- and fs-laser irradiation, respectively. This result substantiates the observation that the single fs pulse laser irradiation appears more effective in inducing crystallization of GST thin films than the single ns pulse laser irradiation. Explanations could be an extreme suppression of the thermal atomic diffusion^34^,^35^ or the occurrence of multiphoton absorption during fs laser irradiation^36^.

## Conclusions

Crystallization of GST thin films deposited by PLD was triggered by UV ns and fs single laser pulses. The phase transition from the amorphous to the crystalline state was studied in dependence on the laser fluence. Crystalline phases could be detected by XRD and TEM after laser ns- and fs-laser irradiation with fluences ≥36 mJ/cm^2^ and ≥13 mJ/cm^2^, respectively. Cross-sectional electron microscopy studies showed a columnar growth of the GST thin films after fs-laser single pulse irradiation whereas oval grains were formed after ns-laser single pulse irradiation. In addition, neither tetrahedral nor off-octahedral positions of Ge-atoms were detected in the defect-free grains after fs-laser irradiation by HRSTEM examination. The optical reflectivity contrast increased with the laser fluence for both ns and fs pulse laser irradiation. A simple model of the irradiation-induced crystalline phase formation was used to assess the average contribution of ns and fs laser photons to the phase transition process. A change of the optical reflectivity contrast of 25% can be obtained by single pulse fs-laser irradiation with a fluence ≥15 mJ/cm^2^ or ns-laser irradiation with a fluence ≥67 mJ/cm^2^. The reflectivity contrast change of 25% corresponds to a complete crystallization of the GST film. The presented results and the calculations based on them allow the conclusion that for the chosen parameters the single fs pulse laser irradiation is more effective in inducing crystallization of GST thin films than the single ns pulse laser irradiation.

## Methods

GST thin films were deposited on Si(100) substrates covered with 500 nm SiO_2_ at room temperature by pulsed laser ablation of a metallic GST alloy target with a chemical composition close to 2:2:5 stoichiometry. A KrF excimer laser was used with a wavelength of 248 nm, pulse duration of 20 ns, and repetition rate of 10 Hz. The laser beam was focused on the target at an incident angle of 60° with respect to the target normal to maintain a laser fluence of 1.5 J/cm^2^. The working pressure was 5 × 10^−6 ^Pa during deposition. The substrates (1 × 1 cm^2^) were ultrasonically cleaned by ethanol and deionized water prior to deposition and were positioned parallel to the target surface at a target-substrate distance of roughly 7 cm inside the vacuum chamber. Both target and substrates were rotated in order to avoid deep ablation of the target and to improve the thickness homogeneity of the films, respectively (details see previous works)^37^,^38^. As-deposited GST films with approximately 90 nm thickness were obtained after PLD with a chemical composition of closely Ge_2_Sb_2_Te_5_ as measured by energy dispersive x-ray analysis.

After deposition, the GST films were irradiated with single UV laser pulses in order to induce an amorphous to crystalline phase transition. To study the influence of the pulse duration on the crystallization process in the GST films, two laser systems at the same wavelength of 248 nm, but with different pulse lengths were applied: on the one hand a ns laser (KrF excimer laser with pulse length of 20 ns) and on the other hand a fs laser (hybrid excimer-dye laser with pulse length of 500 fs). The spot size of the ns rectangularly shaped laser beam was about 24 × 6 mm^2^ with almost a top hat intensity distribution, while the fs laser beam spot size was about 2 × 3 mm^2^. The laser fluence could be varied between 0 and 130 mJ/cm^2^ and between 0 and 19 mJ/cm^2^ for ns laser and fs laser irradiation, respectively.

The optical reflectivity of the GST films was measured by a UV-Vis spectrophotometer in a wavelength range from 400 to 700 nm. The structure analysis of the laser-irradiated films was performed by XRD using Cu Kα radiation in a parallel beam geometry using a 0.11° parallel slit analyzer. Cross-sectional TEM specimens were prepared by using a combination of focused high-energy gallium ion and low-energy argon ion beam milling^29^,^39^,^40^. An ultra-thin coating of electrically conducting platinum metal was used to stabilize the samples during the preparation. Local structure analysis of the GST thin films was carried out by TEM using a FEI Titan^3^ G^2^ 60–300 analytical TEM equipped with a Cs probe corrector and annular dark-field (ADF) detectors. Middle-angle ADF (MAADF) images were taken using annular ranges of 40–200 mrad of HAADF detector whereas low-angle ADF (LAADF) images were recorded using annular ranges of 9.3–53.6 mrad of the ADF detector. A probe forming aperture of 25 mrad was used in the scanning TEM study. The TEM was operated at 300 kV accelerating voltage.

## Additional Information

**How to cite this article**: Sun, X. *et al.* Crystallization of Ge_2_Sb_2_Te_5_ thin films by nano- and femtosecond single laser pulse irradiation. *Sci. Rep.* **6**, 28246; doi: 10.1038/srep28246 (2016).

## Supplementary Material

Supplementary Information
