# Supplementary material for: Crystallization of Ge2Sb2Te5 thin films by nano- and femtosecond single laser pulse irradiation
Source: Sci Rep. 2016 Jun 13;6:28246. doi: 10.1038/srep28246 (PMC4904278; doi:10.1038/srep28246)
Supplement: Supplementary Information [file srep28246-s1.doc]

**Crystallization of Ge_2_Sb_2_Te_5_ thin films by nano- and femtosecond single laser pulse irradiation**

Xinxing Sun^1,*^, Martin Ehrhardt^1^, Andriy Lotnyk^1^, Pierre Lorenz^1^, Erik Thelander^1^, Jürgen W. Gerlach^1^, Tomi Smausz^2^, Ulrich Decker^1^ and Bernd Rauschenbach^1,3^

^1^Leibniz Institute of Surface Modification, Permoserstr. 15, D-04318 Leipzig, Germany

^2^MTA-SZTE Research Group on Photoacoustic Spectroscopy, University of Szeged, Dóm tér 9, H‑6720 Szeged, Hungary

^3^Institute for Experimental Physics II, Leipzig University, Linnéstr. 5, D-04103 Leipzig, Germany

^*^corresponding: xinxing.sun@iom-leipzig.de

**Supplementary Information**

**S1. Simulation of temperature distribution after ns/fs laser irradiation**

To verify the crystalline growth mechanism of GST films correlated with the heat flow condition after nanosecond and femtosecond single pulse laser irradiation, a one and two temperature models^1^ is used, respectively. The finite element method, using the commercial finite element package COMSOL, allows the estimation of the time-dependent temperature distribution in the surface and interface of the films. For ultrashort laser pulses smaller than 10 ps, a disparity of the electron temperature and a lattice temperature is supposed and the laser-solid interaction can be physically described by a two-temperature model^1^.

- Two-temperature model:

Electron temperature (*T_e_*) in GST:

(S1)

Phonon / lattice temperature (*T_p_*) in GST:

(S1)

At short laser pulses, like ns-laser pulses, an equality of the T_e_ and T_p_ can be assumed and a one-temperature model allows a well description of the physical process^1^.

- One-temperature model *(T_p_ = T_e_=T)* in GST:

(S1)

where Q is the laser beam induced heat source, r_s_ is the spatial coordinate (r_s_ = r_s_(x,z), z is the depth, x = distance from the laser spot center), t: time and Δt_p_: laser pulse duration.

| density ρ | 5.5 g·cm^-3^ |
| --- | --- |
| heat capacity at constant pressure c_p_ | 1.55 J·kg^-1^·K^-1^ |
| heat capacity at constant pressure c_p;p/e_ | 1.395/0.155 J·kg^-1^·K^-1^ |
| thermal conductivity κ | 0.27 W·mK^-1^ |
| reflectivity R | 65 % |
| absorption coefficient α | 8×10^5^ cm^-1^ |
| electron-photon coupling strength γ | 10^17^ W/(m^3^∙K) |

**Table S1.** Summary of the assumed GST material parameter^2-5^.

The assumed material parameter of GST is summarized in Table S1. At this first estimation the material parameter are assumed as constant. The material parameter of SiO_2_ and Si was taken from the COMSOL material data base. Further, the thermal diffusivity into the Si and SiO_2_ layer was calculated by a one- temperature model at both cases. As laser beam profile a homogeneous beam profile was assumed. The aim of the model is an estimation of the maximum achievable temperature induced by the laser radiation. Further energy loss mechanism like laser-plasma absorption and melting and evaporation enthalpy were non-regarded. The simulated result of ns and fs single pulse laser irradiation is depicted in Fig. S1.

Figure S1a shows the two-dimensional lattice temperature distribution in a 90 nm GST/500 nm SiO_2_/Si substrate system 50 ps after the single pulse femtosecond laser irradiation with a fluence of 19 mJ/cm^2^, as compare to ns one (see in Figure S1b). The temperature distribution after fs pulse laser irradiation shows a surface-localized heating region, where there is a relatively fast temperature rise and a higher temperature in the top surface of the GST film with a larger temperature gradient through the layer thickness as illustrated in the left of Fig. S1c. Although the lattice temperature increases by about 864 K (591°C) in the top surface, which is much higher than the conventional crystallization temperature (~140°C) for GST film, the crystallization temperature with increasing heating rate has been known to shift to higher temperatures (the measured T_c_ of GST is 356.5° at a heating rate of 4 × 10^4^ K/s) ^6-8^.

In Figure S1c shows the simulation results of electron and lattice temperature by distribution at the surface of the film as a function of time after single fs pulse laser irradiation. The electron temperature immediately rises up to extremely high values (~ above 6500 K) during fs pulse laser irradiation on the GST film, then it quickly cools down (~ 860 K) after 40-50 ps whereas the lattice temperature is gradually increased with increase of the irradiation time and it shows the same temperature range ~ 860 K at the time scale of 50 ps. It is notified that the lattice temperature in the interface increases almost no change (only up to approximately 295 K). For the heating and cooling rate on carrier thermalization, it can be deduced to be about 1.3×10^16^ K/s and 1.7×10^13^ K/s, respectively. For comparison, the simulated temperature distribution as a function of time in a GST film after single pulse ns laser irradiation is shown in the right part of Figure S1c. Moreover, it is found that after single pulse fs laser irradiation within approximately 100 ns room temperature is reached as shown in Figure S1d. In the case of the fs single pulse irradiation, the cooling rate decreases from approx. 1.5 ×10^11^ K/s within the initial 50 ps to 8 ×10^8^ K/s after a delay time of 100 ns, whereas in the case the ns single pulse irradiation, a decrease of the cooling rate from initially 5 ×10^10^ K/s to 4 ×10^8^ K/s after a delay time of 100 ns results (see Figure S1d). The above represented results of the two-temperature model analysis for fs laser irradiation are describing the non-equilibrium process, which is more accurate used than normal thermal diffusion equations. During laser-material interaction, the electrons in the materials are first excited by the deposited laser energy, and these “hot” electrons are then cooled by transferring their energy to the lattice through electron-phonon coupling^9,10^.

**Figure S1.** Modeled two-dimensional temperature distribution of a 90 nm thick GST film on a SiO_2_/Si substrate: **(a)** after single fs pulse laser irradiation with a fluence of 19 mJ/cm^2^ for 50 ps, **(b)** after single ns pulse laser irradiation with a fluence of 36 mJ/cm^2^ for 20 ns. z is the depth, x is the distance from the laser spot center. **(c)** A comparison of temperature - time dependence at the top surface center of the GST film: carrier and lattice temperature as function of time after one fs single pulse at a laser fluence of 19 mJ/cm^2^, (right) after one ns single pulse at a laser fluence of 36 mJ/cm^2^. **(d)** Corresponding comparison of the cooling rates directly after the laser pulse as well as after a delay time of 100 ns for ns and fs single laser pulse irradiation.

**S2. Overview TEM image**

**Figure S2**. Overview aberration-corrected HRSTEM image of a representative fs single laser pulse irradiated GST film viewed along the [001] zone axis. The marked selected area is corresponding to show in Figure 3 (a) MAADF- and (b) LAADF-HRSTEM images, respectively.

**References**

1. Bäuerle, D. *Laser Processing and Chemistry,* 4th Edition Springer Berlin, (Heidelberg, 2011).

2. Lu, H. *et al.* Single pulse laser-induced phase transitions of PLD-deposited Ge_2_Sb_2_Te_5_ films. *Adv. Funct. Mater.* **23,** 3621-3627 (2013).

3. Morales-Sánchez, E., Prokhorov, E., Mendoza-Galván, A. & González-Hernández, J. Determination of the glass transition and nucleation temperatures in Ge_2_Sb_2_Te_5_ sputtered films. *J. Appl. Phys.* **91,** 697 (2002).

4. Lee, J. *et al.* Phonon and Electron Transport through Ge_2_Sb_2_Te_5_ Films and Interfaces Bounded by Metals. *Appl. Phys. Lett.* **102,** 191911 (2013).

# 5. Lee, B. S. *et al.* Investigation of the optical and electronic properties of Ge_2_Sb_2_Te_5_ phase change material in its amorphous, cubic, and hexagonal phases. *J. Appl. Phys.* 97, 093509 (2005).

6. Friedrich, I., Weidenhof, V., Njoroge, W., Franz, P. & Wuttig, M. Structural transformations of Ge_2_Sb_2_Te_5_ films studied by electrical resistance measurements. *J. Appl. Phys.* **87,** 4130 (2000).

7. Kissinger, H.E. Reaction Kinetics in Differential Thermal Analysis. *Anal. Chem.* **29,** 1702 (1957).

# [8.](http://www.sciencedirect.com/science/article/pii/S0921452612003006#bib26) Orava, J., Greer, A. L., Gholipour, B., Hewak, D. W. & Smith, C. E. Characterization of supercooled liquid Ge_2_Sb_2_Te_5_ and its crystallization by ultrafast-heating calorimetry. *Nat. Mater.* 11, 279 (2012).

9. Anisimov, S. I., Kapeliovich, B. L. & Perelman, T. L*.* Electron emission from metal surfaces exposed to ultrashort laser pulses. *Sov. Phys. JETP* **39,** 375 (1974).

10. [Yang](http://www.sciencedirect.com/science/article/pii/S0921510714002839), Q., [Cai](http://www.sciencedirect.com/science/article/pii/S0921510714002839), Z., [Wang](http://www.sciencedirect.com/science/article/pii/S0921510714002839), Y., [Huang](http://www.sciencedirect.com/science/article/pii/S0921510714002839), H. & [Wu](http://www.sciencedirect.com/science/article/pii/S0921510714002839), Y. Controllable crystallization of Ge_2_Sb_2_Te_5_ phase-change memory thin films driven by multiple femtosecond laser pulses. [*Materials Science and Engineering: B*](http://www.sciencedirect.com/science/journal/09215107)  [**193**](http://www.sciencedirect.com/science/journal/09215107/193/supp/C)**,** 189 (2015).
